# Help-Seeking for Sexual Difficulties Among Australian Men: Analysis of the Ten to Men Longitudinal Study

**DOI:** 10.1007/s10508-025-03182-7

**Published:** 2025-07-21

**Authors:** Zelalem Mengesha, Adugnaw Zeleke Alem, Tesfaye Alemayehu Gebremedhin, Alexandra J. Hawkey

**Affiliations:** 1 Health Research Institute, University of Canberra, 11 Kirinari St, Bruce ACT, Canberra, 2617 Australia; 2 Department of Epidemiology and Biostatistics, Institute of Public Health, College of Medicine and Health Sciences, University of Gondar, Gondar, Ethiopia; 3 Canberra School of Politics, Economics & Society, University of Canberra, Canberra, Australia; 4 School of Medicine, Translational Health Research Institute, Western Sydney University, Penrith, Sydney, NSW Australia

**Keywords:** Men, Help-seeking, Sexual health, Sexual difficulties, Sexual dysfunction, Equity

## Abstract

Sexual difficulties are common among Australian men, affecting over half the population regardless of age or sexual identity. It can have significant impacts on physical health, mental well-being, and quality of life if left untreated. The present study aimed to examine sexual difficulty trends, sources of seeking help, and factors associated with sexual help-seeking behavior among a cohort of Australian men. Four waves of data from the Australian Longitudinal Study on Male Health (Ten to Men) were used in the analysis. We included men aged 18 years and above who had engaged in vaginal, oral, or anal sex, leading to a total sample size of 12,737 (wave 1), 8,933 (wave 2), 6,991 (wave 3), and 5,804 (wave 4) men. Logistic regression was undertaken to identify factors associated with sexual help-seeking behavior. Across the four waves, there was a significant rise in the prevalence of men experiencing at least one form of sexual difficulty, increasing from 54.1% (95% CI 52.7, 62.5) in 2013/14 to 64.7% (95% CI 62.8, 66.7) in 2022. The increase in prevalence of sexual difficulties was more pronounced among men from culturally and linguistically diverse backgrounds which increased from 42.8% (95% CI 38.1, 47.5) in 2013/14 to 61.5% (95% CI 55.6, 67.1) in 2022. Around 17.6% of men sought assistance for their sexual health concerns in 2022, with no significant change over the study period. Number of sexual difficulties, age, sexual identity, relationship status, conformity to masculine norms, depression, and number of financial hardships were factors significantly associated with sexual health-seeking behavior. Despite the burden of sexual difficulties increasing among Australian men, few have sought assistance, suggesting a significant level of unmet sexual health need. This underscores the necessity of evaluating current services and considering co-designed sexual health initiatives, informed by an intersectional approach, to enhance accessibility, engagement, and responsiveness to the distinct sexual health needs and preferences of men from a range of communities.

## Introduction

Sexual and reproductive health (SRH) is a fundamental human right essential for mental well-being and overall health (Khosla et al., 2015). The Australian National Men’s Health Strategy 2020–2030 states that SRH is a priority issue needing significant commitment and careful planning of health initiatives and services (Department of Health, 2019). However, SRH research and program development among men in Australia largely focuses on sexually transmitted infections and fertility, meaning other areas of men’s SRH are overlooked (Daumler et al., 2016; Mengesha et al., 2023). For instance, sexual dysfunction, including diminished sexual desire or sexual pleasure, erection and orgasm difficulties, and premature ejaculation (WHO, 2015), is prevalent in the Australian population (Schlichthorst et al., 2016). The Australian Study of Health and Relationships found that approximately half of Australian men reported experiencing at least one sexual difficulty, with lack of interest in sex (28%) and premature ejaculation (21%) being the most frequently reported concerns (Richters et al., 2022). Differences in sexual difficulty among men correlate with health conditions, such as poor self-rated health, disability, and mental health conditions, as well as lifestyle choices, including smoking, harmful alcohol consumption, and drug use (Schlichthorst et al., 2016). This implies that addressing men’s sexual difficulties requires a comprehensive approach that considers both the physical and mental health, and lifestyle aspects of men’s lives (Avasthi et al., 2017; Berry & Berry, 2013).

Sexual difficulties can significantly impact men's physical health, mental well-being, and quality of life (Wagner et al., 2000). For example, erectile dysfunction has been found to contribute to reduced sexual satisfaction (Sheng, 2021). Experiencing higher number of sexual difficulties is linked to psychological distress (Lafortune et al., 2023), including depression, anxiety, and low self-esteem (Mota et al., 2019). Left untreated, these mental health conditions can exacerbate the problem, further inhibiting sexual function and creating a cycle of distress (Xiao et al., 2023). Additionally, unresolved sexual problems may strain intimate relationships leading to communication difficulties, feelings of inadequacy, and relationship breakdowns (Heiman, 2002). Sexual difficulties can also negatively impact overall life satisfaction (Lu et al., 2020) and hinder participation in professional activities posing a substantial economic burden on employers (Elterman et al., 2021). Despite its impact, a significant reluctance among men to seek healthcare professional help persists, making sexual difficulties underreported and undertreated health conditions (Moreira et al., 2005). This suggests that addressing sexual difficulties through timely intervention and support is crucial for promoting men’s SRH and well-being and improving quality of life (McCabe & Althof, 2014).

In Australia, SRH services are provided through a combination of sexual health clinics, general practitioners, family planning organizations, and specialized services, offering care such as contraception, STI prevention and treatment, and fertility-related services. Although a wide range of services exist, access to them is not equitable due to barriers that operate at multiple levels (Parliament of Australia, 2023). At the individual level, men face lack of awareness about available services, language barriers, and confidentiality concerns that discourage them from seeking care for sensitive issues (Mengesha et al., 2023). Interpersonal barriers include challenges in discussing sexual health with partners or peers which again can inhibit help-seeking (Coleman et al., 2019). At the community level, traditional notions of masculinity, which emphasize self-reliance, emotional restraint, and sexual performance, contribute significantly to the stigma surrounding discussions of sexual difficulties and reluctance to access support (Sever & Vowels, 2024). This stigma is further reinforced by cultural and religious norms within Australia’s multicultural population, where discussions about sexual health are often considered taboo (Mengesha et al., 2023). As a result, many men feel embarrassed or ashamed to address intimate concerns, leaving their concerns unaddressed rather than seek assistance from healthcare professionals (Latreille et al., 2014). Structurally, men face barriers to accessing SRH care, including prolonged waiting times and high cost of services (Lafortune et al., 2023). For instance, consultations with a sex therapist or attending couples counseling is often not bulk billed in Australia as these services are not covered by Medicare, creating a financial barrier to access for many individuals (Sever & Vowels, 2024). People living in rural and remote areas also face major challenges due to a shortage of services and the long distances they must travel to reach healthcare providers (Johnston et al., 2015). This implies that comprehensive strategies targeting all levels of barriers are needed to ensure equitable access to SRH services for Australian men.

Existing research into sexual difficulties among men in Australia is frequently confined to cross-sectional studies (Schlichthorst et al., 2016), which poses challenges in comprehending its longitudinal burden. While some research has explored provider perceptions on challenges men encounter in accessing sexual health services (Collyer et al., 2018), few published data investigate sexual help-seeking behavior by men and its determinants (Smith et al., 2013). Drawing on data from the Australian Longitudinal Study on Male Health (Ten to Men), the present study aims to address this gap by analyzing longitudinal trends in sexual difficulty rates, common avenues for seeking help, and factors associated with sexual help-seeking behavior. Understanding factors associated with sexual help-seeking behavior is essential for enhancing equitable access to sexual healthcare, reducing potential stigma, promoting early intervention, and developing programs aimed at improving men’s SRH and psychological well-being (Shand & Marcell, 2021).

## Method

### Participants and Procedure

This analysis was based on data from the Australian Longitudinal Study on Male Health (Ten to Men), a large cohort study of Australian men aged 10–55 at baseline. As described by Currier et al. (2016), a stratified, multistage, random cluster sampling technique was used to examine six key areas of male health: well-being and mental health, use of health services, health-related behaviors, health status, health knowledge, and social determinants. Field workers recruited participants from households in each of the chosen areas if they were males aged 10 to 55, Australian citizens, or permanent residents, and had sufficient English language proficiency to complete a self-administered survey questionnaire. Our analysis utilized the longitudinal data of adult men (age 18 +) across waves 1 (2013–2014), 2 (2015–2016), 3 (2020), and 4 (2022). The 2020 and 2022 waves were collected during the COVID-19 pandemic. The variables of interest were sexual difficulties, help-seeking behavior, and its determinants. In line with previous studies (Schlichthorst et al., 2016), only men who had ever engaged in vaginal, oral, or anal sex were included in our analysis leaving a total sample of 12,737 (wave 1), 8,933 (wave 2), 6,991 (wave 3), and 5,804 (wave 4) men. We restricted our sample to men with prior sexual experience as seven out of the eight measures of sexual difficulties focused on experiences related to intercourse.

### Measures

The key variables of interest in our study were sexual function and sexual help-seeking behavior. In the Ten to Men data, sexual function was assessed using the validated National Survey of Sexual Attitudes and Lifestyles questionnaire (Mercer et al., 2005). The measure comprises eight constructs: lacking interest in having sex, lacking enjoyment in sex, feeling anxious during sex, feeling physical pain because of sex, feeling no excitement or arousal during sex, not being able to reach climax or taking too long, reaching climax too quickly, and having difficulties getting or keeping an erection. Having a binary (yes/no) response option, all sexually active men were asked if they had experienced any of the sexual difficulties for at least three months in the 12 months prior to the study. Sexual help-seeking was measured by asking all sexually active men if they had sought help for sexual health issues from a sexual health clinic, self-help groups, self-help books, relationship counselor, psychiatrist or psychologist, internet, helpline, other type of clinic or doctor, general practitioner (GP)/family doctor, family member/friend 12 months prior to the study. Response options to these questions were binary (yes/no).

Our study controlled for a range of independent variables which were believed to affect men’s sexual help-seeking behavior in the wider literature. These included sociodemographic and economic factors (age, level of education, culturally and linguistically diverse (CALD) status, employment status, area level of disadvantage, sexual identity, relationship status, financial hardship, private health insurance) (Lafortune et al., 2023), health conditions (disability, self-rated general health, depression) (Schlichthorst et al., 2016), geographical factors (Australian Statistical Geography Standard region), and behavioral factors (health literacy, conformity to masculine norms) (Piatkowski et al., 2023).

Conformity to masculine norms: Conformity to Masculine Norms Inventory (CNMI) (Mahalik et al., 2003) was used to measure the degree of conformity to traditional masculine roles. It is comprised of 22 items that capture notions of winning, emotional control, risk taking, violence, dominance, sex, self-reliance, primacy of work, power over women, homosexuality, and pursuit of status with a four-point scale response option: 0 = strongly disagree, 1 = disagree, 2 = agree, and 3 = strongly agree. The scores for each item were summed to produce a total CMNI score, which ranges from 0 to 66, with higher scores indicating greater conformity to masculine norms. The CMNI score is divided into low conformity, moderate conformity, and high conformity according to the quantile. CMNI scores were collected at wave 1 and wave 3 and used as a time-invariant variable at waves 2 and 4.

Severity of depression: A nine item Patient Health Questionnaire (PHQ-9) (Kroenke et al., 2001) was used to assess depression among the Ten to Men participants. The questionnaire scores each of the nine symptoms on a scale of 0 (not at all) to 3 (nearly every day), with the sum of the scores determining the presence and severity of depression. We considered a PHQ-9 score of 1–4, 5–9, 10–14, 15–19, and 20 or more to indicate no/minimal, mild, moderate, moderately severe, and severe depression, respectively.

Health literacy: All participants of the Ten to Men study completed a three-scale Health Literacy Questionnaire (HLQ) (Osborne et al., 2013): ability to find good health information; ability to actively engage with healthcare providers; and feeling understood and supported by healthcare providers (collected only at wave 2). The HLQ comprises several sub-scales that examine the full construct of health literacy with five response options for each item: 0 = “cannot do,” 1 = “very difficult,” 2 = “quite difficult,” 3 = “easy,” and 4 = “very easy.” A score ranging 0–20 was generated for each participant to fit the health literacy variable in the regression model.

CALD: The Australian Bureau of Statistics (ABS) defines CALD as a person who is Non-Aboriginal and/or Torres Strait Islander and speaks a language other than English at home (ABS, 2022). Core variables of CALD standards by the ABS were used to identify CALD men in the Ten to Men study. CALD status was collected at wave 1 and used as a time-invariant variable for waves 2, 3, and 4.

Financial hardship: It was assessed using five (waves 3 and 4) or six (waves 1 and 2) binary (no/yes) items, including could not fill/collect prescription, could not get medical care, could not pay bills on time, could not pay mortgage/rent on time, asked for financial help from others, and/or limited consumption of fruit and vegetables.

### Statistical Analysis

All data management and statistical analyses were performed using Stata version 17 software. Background characteristics of the study participants in each wave were summarized using frequencies and percentages for categorical variables and mean and SDs for continuous variables. The summary statistics for sexual difficulties and sexual health-seeking behavior was compared between CALD and non-CALD men, with 95% confidence intervals (CIs) used to assess the statistical significance of differences across waves. To adjust for non-proportional sampling and non-response rates and obtain robust statistical estimates, data were weighted using the survey estimation commands in Stata (svyset command). In the svyset command, wave specific raked cross-sectional population weight, Statistical Area Level 1 code, and Australian Statistical Geography Standard region were used as variables representing the primary sampling unit, sampling weight, and strata, respectively.

We used logistic regression to investigate potential factors associated with sexual health-seeking behaviors. To select potential variables, we first conducted bivariate logistic regression (unadjusted regression) for each independent variable in each wave. Then, variables with a *p*-value ≤ 0.2 in the bivariate analysis were entered into a multivariate logistic regression model. In multivariate logistic regression, the association between each independent variable and sexual health-seeking behavior was reported using adjusted odds ratios (AORs) and 95% CIs.

## Results

### Background Characteristics

Table 1 shows the background characteristics of the study participants included in the analysis. The distribution of variables was similar across the waves except for age, private insurance, marital status, and number of financial hardships experienced. The proportion of men aged 55 years and over has increased from 2.1% in 2013/14 to 31.4% in 2022. Similarly, the proportion of men with private health insurance increased from 54.5% in 2013/14 to 80.9% in 2022. In 2013/14, more than one in ten men (11.9%) had experienced three or more financial hardships, while this figure decreased to 3.5% in 2022. Most men (82.7% in 2013/14, 82.5% in 2015/16, 81.3% in 2020, and 80.7% in 2022) had moderate conformity to masculine norms.

**Table 1:** Background characteristics of the study participants by year of study

Variables	Wave 1/2013 N = 12,737	Wave 2 /2016 N = 8,933	Wave 3 /2020 N = 6,991	Wave 4 /2022 N = 5,804
	N (%)	N (%)	N (%)	N (%)
Age (in years)				
18–24	1,532 (12.0)	851 (9.5)	558 (8.0)	375 (6.4)
25–34	2,895 (22.7)	1,754 (19.6)	969 (13.9)	713 (12.3)
35–44	3,942 (31.0)	2,669 (29.9)	1,536 (22.0)	1,160 (20.0)
45–54	4,106 (32.2)	2,940 (32.9)	2,205 (31.5)	1,734 (29.9)
≥ 55	262 (2.1)	719 (8.1)	1,723 (24.6)	1,822 (31.4)
Employment status				
Employed	10,990 (87.0)	8,075 (90.5)	6,159 (89.3)	5,160 (89.7)
Unemployed	1,648 (13.0)	845 (9.5)	738 (10.7)	589 (10.3)
Highest level of education achieved	**	**		
University degree			2,133 (40.0)	1,967 (34.2)
Certificate/diploma			3,068 (57.6)	2,478 (43.1)
Year 12 or less			125 (2.4)	1,302 (22.7)
SEIFA index				
High disadvantage	4,978 (39.1)	2,221 (25.0)	2,214 (33.7)	1,721 (30.8)
Middle disadvantage	4,725 (37.1)	3,491 (39.4)	2,249 (34.3)	1,766 (31.6)
Low disadvantage	3,032 (23.8)	3,162 (35.6)	2,103 (32.0)	2,101 (37.6)
CALD status				
Non-CALD	11,374 (91.6)	7,918 (90.5)	5,990 (89.9)	4,967 (86.7)
CALD	1,036 (8.4)	833 (9.5)	899 (13.1)	760 (13.3)
Private insurance				
No	5,640 (45.5)	3,487 (39.5)	2,598 (38.3)	869 (19.1)
Yes	6,764 (54.5)	5,334 (60.5)	4,181 (61.7)	3,689 (80.9)
Region				
Remote	-	13 (0.2)	25 (0.4)	22 (0.4)
Regional	5,285 (41.5)	3,551 (40.0)	2,454 (37.3)	2,112 (37.7)
Major cities	7,450 (58.5)	5,314 (59.8)	4,097 (62.3)	3,473 (61.9)
Sexual identity		**		
Heterosexual	11,790 (94.1)		6,455 (93.8)	5,417 (93.9)
Bisexual	181 (1.5)		220 (3.2)	169 (2.9)
Gay	206 (1.6)		182 (2.6)	150 (2.6)
Others*	347 (2.8)		24 (0.4)	31 (0.6)
Disability				
No	11,720 (93.1)	8,290 (93.3)	6,566 (94.1)	5,440 (93.9)
Yes	871 (6.9)	597 (6.7)	411 (5.9)	355 (6.1)
Severity of depression				
No/minimal	7,962 (63.9)	5,953 (67.4)	4,487 (64.6)	3,718 (64.2)
Mild	2,898 (23.2)	1,939 (22.0)	1,563 (22.5)	1,340 (23.1)
Moderate	988 (7.9)	597 (6.8)	513 (7.4)	440 (7.6)
Moderately severe	401 (3.2)	235 (2.6)	242 (3.5)	189 (3.3)
Severe	221 (1.8)	106 (1.2)	137 (2.0)	104 (1.8)
Self-rated health	**	**		
Excellent			865 (12.4)	612 (10.6)
Very good			2,754 (39.4)	2,193 (37.8)
Good			2,444 (35.0)	2,206 (38.0)
Fair/poor			926 (13.2)	792 (13.6)
Relationship status				
Single/separated/widowed/divorced	3,817 (30.2)	1,841 (20.7)	1,058 (15.2)	893 (15.5)
Married or in a de-facto relationship	8,805 (69.8)	7,037 (79.3)	5,884 (84.8)	4,887 (84.5)
Conformity to Masculine Norms				
Low	2,039 (16.9)	1,454 (17.2)	1,235 (18.5)	1,059 (19.1)
Middle	9,939 (82.7)	6,966 (82.5)	5,422 (81.3)	4,474 (80.7)
High	48 (0.4)	27 (0.3)	14 (0.2)	11 (0.2)
Number of financial hardships				
0 (no)	8,579 (68.2)	6,651 (74.9)	5,862 (85.1)	4,917 (85.5)
One	1,539 (12.2)	988 (11.1)	563 (8.2)	438 (7.6)
Two	975 (7.7)	587 (6.6)	227 (3.3)	196 (3.4)
Three or more	1,495 (11.9)	653 (7.4)	233 (3.4)	198 (3.5)
Health literacy: Ability to actively engage with services (M ± SD)	**	14.67 ± 3.68	16.01 ± 3.44	16.25 ± 3.51
Health literacy: Ability to find good health information (M ± SD)	**	14.90 ± 3.39	15.90 ± 3.26	16.17 ± 3.25

^*^ Asexual, pansexual, and queer, ^**^ Data not collected/high missing values

### Trends in Sexual Difficulties

**Fig. 1:**
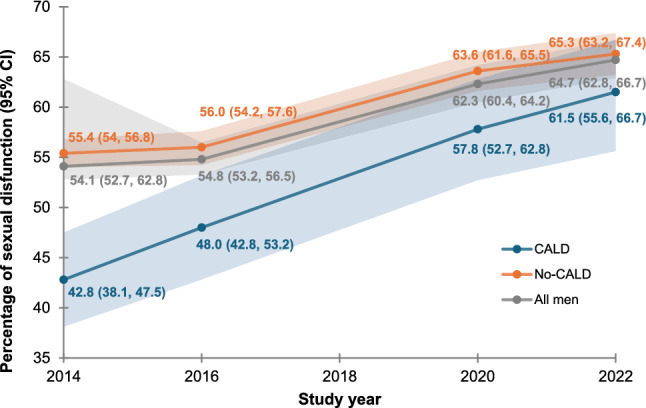
Trends in sexual difficulty among Australian men (2013 to 2022)

As shown in Fig. 1, the prevalence of having at least one sexual difficulty increased significantly, from 54.1% (95% CI: 52.7, 62.8) in 2013/14 to 64.7% (95% CI: 62.8, 66.7) in 2022. The increase was more pronounced among CALD men, from 42.8% (95% CI: 38.1, 47.5) in 2013/14 to 61.5% (95% CI: 55.6, 67.1) in 2022. It is also apparent from Table 2 that the prevalence of each type of sexual difficulty increased significantly between 2013/14 and 2022. Reaching climax too early was the most common form of sexual difficulty in all waves (36.2%, 95% CI: 34.8,37.5 in 2013/14, 36.9%, 95% CI: 35.2,38.5 in 2015/16, 40.1%, 95% CI: 38.3,41.9 in 2020 and 41.0%, 95% CI: 38.9,43.1 in 2022), followed by lacked interest in having sex (17.5%, 95% CI: 16.4,18.6 in 2013/14, 17.7%, 95% CI: 16.5, 19.0 in 2015/16, 24.2%, 95% CI: 22.6, 25.8 in 2020, and 25.4%, 95% CI: 23.6,27.4 in 2022). On average, men reported having 1.12 sexual difficulties (M = 1.12; 95% CI: 1.08–1.17) in 2013/14 and 1.51 (M = 1.51, 95% CI: 1.44,1.58) in 2022.

**Table 2:** Prevalence of each domain of sexual difficulty by year of study

Domains	2013/Wave 1	2016/Wave 2	2020/Wave 3	2022/Wave 4
	%(95% CI)	%(95% CI)	%(95% CI)	%(95% CI)
Lacked interest in having sex	17.5(16.4,18.6)	17.7(16.5,19.0)	24.2(22.6,25.8)	25.4(23.6,27.4)
Lacked enjoyment in sex	10.4(9.6,11.3)	12.3(11.1,13.5)	13.2(12.0,14.6)	15.3(13.7,17.0)
Felt anxious during sex	11.4(10.4,12.4)	13.3((12.1,14.5)	16.1(14.8,17.5)	18.0(16.3,19.8)
Felt physical pain as a result of sex	3.6(3.1,4.2)	3.9(3.3,4.6)	4.7(3.9,5.6)	5.5(4.6,6.7)
Felt no excitement or arousal during sex	5.9(5.2,6.6)	7.8(6.9,8.9)	10.2(9.1,11.4)	10.3(8.9,11.8)
Did not reach climax or took a long time	15.4(14.4,16.5)	17.7(16.5,19.0)	23.0(21.5,24.6)	22.6(20.9,24.4)
Reached climax too quickly	36.2(34.8,37.5)	36.9(35.2,38.5)	40.1(38.3,41.9)	41.0(38.9,43.1)
Had trouble getting or keeping an erection	13.5(12.6,14.5)	15.4(14.2,16.6)	22.9(21.4,24.5)	25.3(23.5,27.2)
Number of sexual difficulties (M, 95% CI))	1.12(1.08, 1.17)	1.25(1.19,1.30)	1.51(1.44,1.58)	1.51(1.44,1.58)
0	45.9(44.5,47.2)	45.2(43.5,46.8)	37.7(35.8,39.6)	35.3(33.2,37.3)
1	27.1(25.8,28.4)	24.7(23.3,26.1)	25.7(24.1,27.3)	26.7(25.0,28.6)
2	12.0(11.2,12.9)	12.3(11.3,13.4)	14.1(12.8,15.5)	15.5(14.1,17.0)
3	6.7(6.1,7.4)	6.9(6.1,7.7)	8.6(7.7,9.6)	9.5(8.4,10.9)
4	3.5(3.0,4.1)	4.7(4.0,5.5)	5.3(4.6,6.2)	5.5(4.6,6.5)
5	2.5(2.1,2.9)	3.2(2.5,3.9)	3.7(3.0,4.6)	3.6(2.9,4.6)
6	1.5(1.2,1.8)	2.1(1.6,2.7)	2.9(2.3,3.5)	2.4(1.9,3.1)
7	0.6(0.4,0.8)	0.7(0.5,1.0)	1.6(1.2,2.2)	1.2(0.8,1.7)
8	0.2(0.1,0.4)	0.3(0.1,0.5)	0.3(0.2,0.6)	0.2(0.1,0.3)

### Prevalence of Sexual Difficulties by Background Characteristics

Men with mild to severe depression have a significantly higher prevalence of sexual difficulties compared to those with no or minimal depression in all waves. For example, the prevalence of sexual difficulties among men with severe depression was 83.3% in wave 1, 90.0% in wave 2, 82.0% in wave 3, and 86.7% in wave 4, compared to 46.0% in wave 1, 46.4% in wave 2, 55.2% in wave 3, and 57.5% in wave 4 among those with no or minimal depression. Men experiencing three or more financial hardships have a significantly higher prevalence of sexual difficulties compared to those with no financial hardships in all waves. Moreover, men with disabilities have a higher prevalence of sexual difficulties compared to those without disabilities in all waves. The prevalence of sexual difficulties also increased with age, as shown in Table 3.

**Table 3:** Prevalence of at least one sexual difficulty according to background characteristics of participants

Variables	Proportion of at least one sexual difficulty							
	Wave 1	χ^2^	Wave 2	χ^2^	Wave 3	χ^2^	Wave 4	χ^2^
	Yes		Yes		Yes		Yes	
Age (in years)								
18–24	47.8	< 0.001	52.6	0.045	58.9	0.650	65.7	0.680
25–34	52.8		51.2		61.6		65.8	
35–44	54.5		57.5		63.8		66.6	
45–54	58.3		55.9		62.7		63.3	
≥ 55	58.6		58.0		63.0		62.8	
Employment status								
Employed	53.6	0.161	53.7	0.006	61.9	0.064	64.5	0.640
Unemployed	56.9		64.1		67.7		66.1	
Highest level of education achieved	**		**					
University degree					58.5	0.029	61.1	0.063
Certificate/diploma					64.4		66.7	
Year 12 or less					59.5		65.3	
SEIFA index								
High disadvantage	54.5	0.253	54.5	0.079	61.9	0.386	64.5	0.737
Middle disadvantage	55.1		52.8		64.0		63.7	
Low disadvantage	52.5		57.0		61.0		65.5	
Private insurance								
No	55.8	0.072	56.7	0.134	63.3	0.500	75.2	< 0.001
Yes	53.0		54.1		62.0		63.7	
Region								
Remote		0.311	61.1	0.848	75.0	0.575	59.8	0.839
Regional	55.0		55.1		62.4		65.4	
Major cities	53.8		54.7		62.2		64.3	
Sexual identity			**					
Heterosexual	53.9	0.258			61.6	< 0.001	63.9	0.003
Bisexual	64.1				74.2		76.0	
Gay	60.9				72.8		72.8	
Others	50.0				93.6		92.2	
Disability								
No	52.8	< 0.001	53.3	< 0.001	61.5	0.003	63.8	0.007
Yes	71.4		76.4		73.9		75.9	
Severity of depression								
No/minimal	46.0	< 0.001	46.4	< 0.001	55.2	< 0.001	57.5	< 0.001
Mild	64.6		66.4		71.5		71.3	
Moderate	69.7		78.2		72.4		82.8	
Moderately severe	79.4		83.9		82.8		81.1	
Severe	83.3		90.0		82.0		86.7	
Self-rated health	**		**					
Excellent					52.3	< 0.001	52.0	< 0.001
Very good					58.9		60.1	
Good					66.2		67.1	
Fair/poor					71.3		80.0	
Relationship status								
Single/separated/widowed/divorced	52.3	0.107	56.3	0.304	60.8	0.442	63.8	0.653
Married or in a de-facto relationship	55.1		54.3		62.8		65.1	
Conformity to Masculine Norms								
Low	53.3	0.761	54.4	0.445	62.2	0.896	65.8	0.816
Middle	54.6		55.1		62.8		64.8	
High	59.1		73.4		68.6		74.0	
Number of financial hardships								
0 (no)	49.7	< 0.001	51.6	< 0.001	60.0	< 0.001	62.5	< 0.001
One	57.4		58.9		72.3		70.6	
Two	63.8		63.6		71.6		75.5	
Three or more	69.5		76.6		78.1		83.9	

Others: Asexual, pansexual, and queer, x^2^: chi-square *p*-value, ** Data not collected/high missing values

### Trends of Sexual Help-Seeking Behavior

**Fig. 2:**
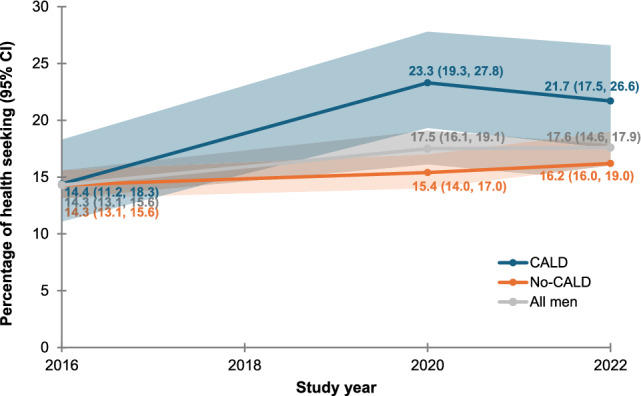
Trends in sexual health-seeking behavior among Australian men (2013 to 2022)

Sexual help-seeking behavior of Australian men remained low over the study period, although there was a significant improvement from 14.3% (95% CI: 13.1, 15.6) in 2015/16 to 17.6% (95% CI: 14.6, 17.9) in 2022. Sexual health-seeking behavior among CALD men also improved significantly from 14.4% (95% CI: 11.2, 18.3) in 2015/16 to 23.3% (95% CI: 19.3, 27.8) in 2020 but declined between 2020 and 2022 (Fig. 2).

### Sources of Help for Sexual Health

**Fig. 3:**
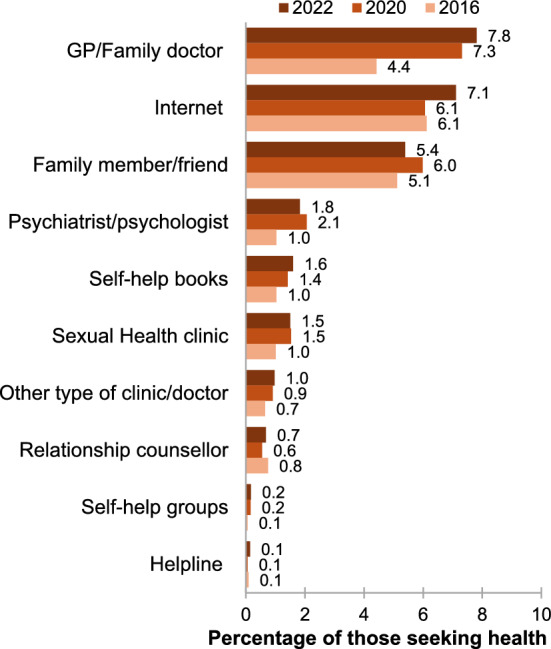
Sources help for sexual health among Australian men by year of study

The primary sources of assistance for sexual health issues in 2020 and 2022 were GPs/family doctors (7.81% and 7.31%) and the internet (7.11% and 6.06%). In 2015/16, the predominant source of help was the internet (6.12%), followed by family or friends (5.12%) (Fig. 3).

### Factors Associated with Sexual Help-Seeking Behavior

The results from the estimation of the logistic regression model, as shown in Table 4, indicate that after adjusting for potential confounders, number of sexual difficulties, age, sexual identity, relationship status, conformity to masculine norms, depression, and number of financial hardships experienced were significantly associated with sexual health-seeking behavior. Specifically, number of sexual difficulties experienced, and age were significantly associated with sexual health-seeking behavior in all waves.

**Table 4:** Factors associated with health-seeking behavior for sexual health among Australian men

Variables	Wave 2/2016	Wave 3/2020	Wave 4/2023
	AOR (95% CI)	AOR (95% CI)	AOR (95% CI)
*Age (in years)*			
18–24	1	1	1
25–34	0.82(0.57,1.19)	0.56(0.30,1.06)	0.59(0.30, 1.18)
35–44	0.57(0.40,0.82)*	0.42(0.22,0.79)*	0.68(0.34, 1.36)
45–54	0.50(0.35,0.73)**	0.36(0.20,0.67)*	0.48(0.24,0.97)*
≥ 55	0.65(0.39,1.06)	0.42(0.22,0.79)*	0.76(0.37, 1.57)
*Number of sexual difficulties*			
0	1	1	1
1–2	1.71(1.36,2.14)**	2.29(1.63,3.23)**	2.06(1.47,2.90)**
≥ 3	4.85(3.67,6.41)**	6.37(4.48,9.03)**	4.75(3.36,6.73)**
*Severity of depression*			
No/miminal	1	1	1
Mild	1.24(0.97,1.60)	1.09(0.81,1.46)	1.33(0.98,1.81)
Moderate	1.80(1.24,2.61)*	1.18(0.80,1.74)	0.91(0.57,1.45)
Moderately severe	1.06(0.63,1.80)	1.28(0.74,2.21)	0.92(0.45,1.85)
Severe	1.48(0.76,2.91)	1.17(0.57,2.42)	1.37(0.61,3.12)
*Sexual identity *	NC		
Heterosexual		1	1
Bisexual		1.86(1.14,3.02)*	2.02(1.18,3.48)*
Gay		3.66(2.08,6.44)**	2.14(0.99,4.61)
Others		3.07(0.62,15.16)	1.22(0.38, 3.90)
*Disability*			
No	1	1	1
Yes	1.04(0.72,1.50)	1.71(1.12,2.63)*	1.58(0.93,2.72)
*Relationship status*			
Single/separated/widowed/divorced	1	1	1
Married or in a de-facto relationship	0.65(0.51,0.84)*	0.74(0.54,1.02)	0.76(0.53,1.09)
*Conformity to Masculine Norms*	NBR	NBR	
Low			1
Middle			0.94(0.66,1.34)
High			6.19(1.38,27.74)*
*Number of financial hardships*			
0 (no)	1	1	1
One	1.20(0.89,1.63)	1.39(0.93,2.07)	2.03(1.36,3.01)**
Two	1.02(0.69,1.53)	1.52(0.91,2.55)	2.61(1.49,4.58)*
Three or more	1.44(1.04,2.02)*	1.62(0.95,2.75)	2.24(1.23,4.06)*

Note: * *p*-value < 0.05; ** *p*-value < 0.001, NBR: Not selected by binary regression, NC: Data not collected/high missing values, others: Asexual/Pansexual/Queer, CI: Confidence interval, AOR: Adjusted odds ratio

In our estimation, number of sexual difficulties experienced had the largest effect on health-seeking behavior in terms of magnitude. For example, the odds of seeking sexual health services in 2020 were 6.37 (AOR = 6.37, 95% CI: 4.48, 9.03) times higher for those who had three or more sexual difficulties compared to those who did not have any difficulties. The corresponding adjusted odds ratios for 2015/16 and 2022 were 4.85 (AOR = 4.85, 95% CI: 3.67, 6.41) and 4.75 (AOR = 4.75, 95% CI: 3.36, 6.73), respectively. Moreover, the results indicate younger men in the 18–24 age category were more likely to seek help for sexual difficulties compared to men in older age groups. For example, the odds of sexual health-seeking among men aged 45–54 was lower by 50% (AOR = 0.50, 95% CI: 0.35, 0.73) in 2015/16, 64% (AOR = 0.36, 95% CI: 0.20, 0.67) in 2020 and 52% (AOR = 0.48, 95% CI: 0.24, 0.97) in 2022 compared to men aged 18- 24 years. Our results found that bisexual and gay men also had higher odds of seeking sexual health services than heterosexual men. Bisexual men had 1.86 (AOR = 1.86, 95% CI: 1.14, 3.02) and 2.02 (AOR = 2.02, 95% CI: 1.18, 3.48) times higher odds of sexual health-seeking than heterosexual men in 2020 and 2022, respectively. The odds of sexual health-seeking among gay men was higher by a factor of 3.66 (AOR = 3.66, 95% CI: 2.08, 6.44) compared to heterosexual men in 2022.

Similarly, the results indicate higher odds of accessing sexual health services for men who experienced one, two, and three or more financial difficulties compared to those who did not experience any financial hardship. We also found that men in a married or de-facto relationship had lower odds of seeking sexual health services compared to those who were single/separated/widowed/divorced (significant only in 2015/16). Lastly, other factors which significantly increased the likelihood that men sought help included moderate depression (significant in 2015/16), disability (significant in 2020), and high conformity to masculine norms (significant in 2022).

## Discussion

Using data from the Ten to Men study, we examined the longitudinal burden of sexual difficulties, common sources for seeking help, and factors associated with sexual help-seeking behavior among a cohort of Australian men. Our findings indicate a significant rise in the prevalence of experiencing at least one sexual difficulty among all men, increasing from 54.1% in 2013/14 to 64.7% in 2022. Particularly notable increases were observed among men from CALD backgrounds, increasing from 42.8% in 2013/14 to 61.5% in 2022. Despite the high prevalence of sexual difficulties among Australian men, we found only about one-fifth sought sexual health assistance with no longitudinal changes over time. The analysis also identified that number of sexual difficulties, age, sexual identity, relationship status, conformity to masculine norms, depression, and number of financial hardships were factors significantly associated with sexual health-seeking behavior.

The increasing rates of sexual difficulties among Australian men may be attributed to the increasing age of the study population, with the proportion of those over 55 years old rising from 2.1 to 31.3% over the study period. Comparing the results with previous studies reveals similar trends in the prevalence of sexual difficulties. For example, a study by Lewis et al. (2004) found a comparable increase in sexual difficulty rates among men in Western countries, pointing to aging populations, lifestyle changes, and increased awareness and reporting of sexual health concerns as contributing factors. The higher rates of increases in sexual difficulties observed among CALD men align with studies that highlight disparities in sexual health outcomes among diverse populations (Mengesha et al., 2023). It underscores the growing impact of sexual health concerns on men’s well-being and highlights the importance of addressing them as a public health priority.

Despite the high prevalence of sexual difficulties among Australian men, we found only about one-fifth sought sexual health assistance with no significant longitudinal changes over the study period. Sever and Vowels (2023) explained that psychological barriers such as embarrassment, shame, and stigma, lack of awareness around where to find sex therapy, and a perception that sexual concerns are not severe health problems hinder the utilization of sexual health services. In Australia, specialized sex therapies and counseling are mostly not covered by Medicare, which disproportionately affects vulnerable populations, including CALD men who are typically from lower socioeconomic backgrounds (Mengesha et al., 2023), highlighting the need for policy reforms to ensure equitable access to sexual health services (Sever & Vowels, 2024). This is particularly critical given that Australia’s Men’s Health Strategy 2020–2030 has identified CALD men as a priority population and SRH as a priority area for intervention to enhance men’s health and well-being outcomes (Department of Health, 2019).

The finding that GPs, the internet, and family members are the most reported sources of help for addressing sexual health concerns has significant implications. It underscores the crucial role of GPs in providing accessible and reliable sexual health services, highlighting the need for ongoing training and resources to ensure they can offer comprehensive support (Ramanathan & Redelman, 2020). This involves ensuring they have current knowledge on diagnosing and treating sexual dysfunction as well as effective communication techniques to sensitively address men’s sexual health issues (Collyer et al., 2018). In addition, men’s reliance on the internet suggests a need for reliable and evidence-based online resources about sexual difficulties (Zarski et al., 2022). The involvement of family members also emphasizes the importance of fostering open and informed discussions about sexual health within families, particularly in marginalized communities (WHO, 2010). These insights can guide targeted interventions and educational efforts to improve sexual health support across these key channels.

Our analysis also revealed that men who experienced financial hardships were more likely to seek help for sexual health issues. This finding can be attributed to the significantly higher prevalence of sexual difficulties among men facing financial hardship, as financial stress negatively impacts both mental and physical health, thereby increasing the likelihood of experiencing sexual difficulties. In turn, we found that men with three or more sexual difficulties had significantly higher odds of seeking sexual health services compared to those without any difficulties. This suggests that financial hardships contribute to higher rates of sexual difficulties, which, in turn, strongly motivate men to seek support for sexual health issues, potentially overriding financial barriers. Additionally, individuals experiencing financial hardship may qualify for subsidized or free healthcare services through the concession healthcare card system in Australia (Zhou & Du, 2023), further reducing cost-related barriers and facilitating access to care. These findings highlight the interconnected relationship between financial hardship, sexual difficulties, and help-seeking behaviors, emphasizing the importance of targeted interventions to address these challenges.

Overall, the significant increase in sexual difficulties among CALD men and the identified variations in sexual help-seeking behavior by age, sexual identity, relationship status, and number of sexual difficulties suggest the need to address how these intersecting factors influence sexual healthcare and related health outcomes (Bowleg et al., 2013). By adopting intersectionality—a framework that examines how overlapping identities such as age, CALD status, sexual identity, and socioeconomic status interact with systemic inequalities—researchers and healthcare professionals can better understand the multifaceted nature of men’s experiences with sexual health (Griffith, 2018). Research shows that marginalized men often face compounded barriers, including stigma, discrimination, and inadequate access to culturally responsive care (Willis & Porche, 2006). Integrating intersectional approaches into healthcare service design and delivery can help reduce these barriers, improve engagement, and promote equity (Mothupi et al., 2023).

Given the increasing prevalence of sexual health issues among Australian men, there is a need for further research to understand the factors underlying the rise in sexual difficulties. Special consideration should be given to CALD men who reported a notably higher increase in rates of sexual difficulties compared to other groups of Australian men. Qualitative studies involving men and sexual health providers are also needed to allow for a more comprehensive understanding of men’s journey toward sexual health services, including the underlying barriers and motivations (Shand & Marcell, 2021).

The study’s strength lies on the use of the four waves of Australia’s Longitudinal Study on Male Health to examine the burden of sexual difficulties over a ten-year period. This is significant as there has been little research examining sexual difficulty rates and help-seeking using longitudinal data. Furthermore, the study has provided additional insights into the emerging research interest of sexual help-seeking for sexual difficulties and factors associated with help-seeking behavior. The Ten to Men study only included participants who had sufficient English language proficiency to complete the survey. Consequently, men from CALD backgrounds who lacked English proficiency were excluded, which may limit the generalizability of the results of our analysis to all CALD men in Australia. In our logistic regression analysis aimed at identifying factors associated with sexual help-seeking behavior, we treated the three waves as cross-sectional data. In future analyses of the Ten to Men dataset, we plan to conduct a more complex analysis to explore the longitudinal associations of health, lifestyle, behavioral, and socioeconomic factors with sexual difficulties and help-seeking behavior. The other notable limitation of this study is the treatment of help-seeking behavior as a binary outcome which may oversimplify the diverse range of actions and motivations involved. Future research should adopt more detailed measures to better reflect the complexity of health-seeking practices.

Additionally, the original Ten to Men study did not collect data on the severity of symptoms and distress related to the eight constructs of sexual difficulties. Data on relational factors such as relationship quality, partner characteristics, partner attraction, and relationship duration were also not captured. These relational dynamics are critical determinants of sexual experiences and could significantly influence the presence or perception of sexual difficulties reported by men. This limitation restricts our ability to understand whether the reported sexual difficulties indicate a medical or psychological condition requiring treatment or a defensive reaction to relational challenges. For instance, Bancroft et al. (2003) demonstrated the importance of considering relational factors when examining sexual distress, highlighting the interplay between these dynamics and sexual health outcomes. Therefore, future iterations of the Ten to Men study should consider including measures that assess the severity of the difficulties, the quality and characteristics of relationships, as well as relational contexts. Such data would enable a more nuanced analysis of sexual difficulties by distinguishing between individual-level factors and those rooted in relational or contextual dynamics. These limitations also underscore the importance of interpreting “help-seeking behavior” within a broader framework that considers the self-reported nature of sexual difficulties and relational and contextual dimensions influencing sexual experiences. By expanding the scope of data collection, future studies could better inform targeted interventions and provide a more holistic understanding of men’s sexual health.

### Conclusions

The present study expanded the current literature on Australian men’s sexual difficulties and help-seeking by examining longitudinal changes and factors associated with help-seeking behavior. Our analysis revealed a significantly increasing burden of sexual difficulties, with CALD men experiencing higher increases compared to other Australian men. Moreover, most men with sexual difficulties do not seek help for them which suggests a high level of unmet sexual health needs. Number of sexual difficulties, age, sexual identity, relationship status, conformity to masculine norms, depression, and number of financial hardships were significantly associated with sexual health-seeking behavior. This suggests the necessity of applying an intersectional lens into the design of programs that aim to improve men’s sexual help-seeking behavior.

## Data Availability

Readers interested in the data for the current study are invited to contact the Ten to Men study team.
