# Correction: Help-Seeking for Sexual Difficulties Among Australian Men: Analysis of the Ten to Men Longitudinal Study

**DOI:** 10.1007/s10508-025-03294-0

**Published:** 2025-09-02

**Authors:** Zelalem Mengesha, Adugnaw Zeleke Alem, Tesfaye Alemayehu Gebremedhin, Alexandra J. Hawkey

**Affiliations:** 1 Health Research Institute, University of Canberra, 11 Kirinari St, Bruce ACT, Canberra, 2617 Australia; 2 Department of Epidemiology and Biostatistics, Institute of Public Health, College of Medicine and Health Sciences, University of Gondar, Gondar, Ethiopia; 3 Canberra School of Politics, Economics & Society, University of Canberra, Canberra, Australia; 4 School of Medicine, Translational Health Research Institute, Western Sydney University, Penrith, Sydney, NSW Australia

**Correction to: Archives of Sexual Behavior** 10.1007/s10508-025-03182-7

The term “sexual dysfunction” should have read “sexual difficulty” in the caption for Fig. 1 and in Table 2 in the article as originally published.

The original article has been corrected.

